# Basal Dendrites of Layer-III Pyramidal Neurons do not Scale with Changes in Cortical Magnification Factor in Macaque Primary Visual Cortex

**DOI:** 10.3389/fncir.2016.00074

**Published:** 2016-09-21

**Authors:** Tomofumi Oga, Tsuguhisa Okamoto, Ichiro Fujita

**Affiliations:** ^1^Graduate School of Frontier Biosciences, Osaka UniversitySuita, Osaka, Japan; ^2^Center for Information and Neural Networks (CiNet), National Institute of Information and Communications Technology and Osaka UniversitySuita, Osaka, Japan

**Keywords:** retinotopic maps, macaque monkey, spines, pyramidal cell, dendrites, visual eccentricity, cortical magnification factor

## Abstract

Neurons in the mammalian primary visual cortex (V1) are systematically arranged across the cortical surface according to the location of their receptive fields (RFs), forming a visuotopic (or retinotopic) map. Within this map, the foveal visual field is represented by a large cortical surface area, with increasingly peripheral visual fields gradually occupying smaller cortical areas. Although cellular organization in the retina, such as the spatial distribution of ganglion cells, can partially account for the eccentricity-dependent differences in the size of cortical representation, whether morphological differences exist across V1 neurons representing different eccentricities is unclear. In particular, morphological differences in dendritic field diameter might contribute to the magnified representation of the central visual field. Here, we addressed this question by measuring the basal dendritic arbors of pyramidal neurons of layer-IIIC and adjoining layer III sublayers (in the Hassler’s nomenclature) in macaque V1. We labeled layer-III pyramidal neurons at various retinotopic positions in V1 by injecting lightly fixed brain tissue with intracellular dye, and then compared dendritic morphology across regions in the retinotopic map representing 0–20° of eccentricity. The dendritic field area, total dendritic length, number of principal dendrites, branching complexity, spine density and total number of spines were all consistent across different retinotopic regions of V1. These results indicate that dendrites in layer-III pyramidal neurons are relatively homogeneous according to these morphometric parameters irrespective of their locations in this portion of the retinotopic map. The homogeneity of dendritic morphology in these neurons suggests that the emphasis of central visual field representation is not attributable to changes in the basal dendritic arbors of pyramidal neurons in layer III, but is likely the result of successive processes earlier in the retino-geniculo-striate pathway.

## Introduction

In the mammalian primary visual cortex (V1), visual information from the left and right visual fields is processed in the contralateral hemisphere. Across the cortical sheet of each hemisphere, neurons are systematically arranged according to the location of their receptive field (RF). Nearby neurons in the cortex respond to visual inputs originating from nearby locations in the visual field (i.e., in the retina) forming a visuotopic (retinotopic) map. The retinotopic map was independently discovered by Inouye (1909); (translated in Glickstein and Fahle, 2000) and Holmes and Lister (1916) by analyzing the spatial relationship between visual field deficits and the gunshot path through the skull of wounded soldiers. As these pioneering studies already noticed and later studies on human and animals detailed, the foveal visual field is represented by a large cortical surface area, while gradually smaller areas are allocated to more peripheral visual fields (Daniel and Whitteridge, 1961; Gattass et al., 1981, 1987; Tootell et al., 1982; Van Essen et al., 1984; Fritsches and Rosa, 1996).

How much cortex is devoted to a given visual field can be quantified by the cortical magnification factor, which is defined as cortical surface area divided by the size of visual field represented in it (mm^2^/deg^2^; Talbot and Marshall, 1941; Daniel and Whitteridge, 1961). In human V1, the magnification factor has been estimated to be 16 mm^2^/deg^2^ at 2° and 0.25 mm^2^/deg^2^ at 25° (Cowey and Rolls, 1974), and in squirrel monkeys, it was shown to be 54.4 mm^2^/deg^2^ for the foveal field (0°), 31.5 mm^2^/deg^2^ at 0.5°, 9.6 mm^2^/deg^2^ at 2°, 0.10 mm^2^/deg^2^ at 20° and 0.01 mm^2^/deg^2^ at 50° (Adams and Horton, 2003). The gradual decrease in cortical magnification factor as visual fields move toward the periphery can be partially explained by the fovea-centric distribution pattern of ganglion cells in the retina. For example, in squirrel monkeys the density of retinal ganglion cells (RGCs) in the foveal region (0–1°) is five times higher than in the near periphery (2–4°) and 235 times higher than in the far periphery (50–71°; Adams and Horton, 2003). If the amount of divergent and convergent retinal projections to V1 is constant across visual eccentricities, the cortical magnification factor should decrease from center to periphery at the same rate as the RGC density. Assuming this linear relationship, the cortical magnification factor in the squirrel monkey fovea (at 0.5°) should be about 235 times larger than that in the periphery (at 50°). However, it is actually 3,150 times larger (31.5/0.01), which is much greater than expected. Thus, changes in cortical magnification factor roughly parallel those in RGC density, but full explanation needs further neuronal mechanism that amplifies the emphasis of the central visual field (Myerson et al., 1977; Schein and de Monasterio, 1987).

Chaplin et al. (2013) suggest that the retina–lateral geniculate nucleus (LGN)–V1 projections are more divergent for central visual fields than for peripheral visual fields. They estimated the area activated by a single point of light, point image size (Capuano and McIlwain, 1981) of V1 in marmoset monkeys by mapping the retinotopy with electrophysiological techniques accurately on distortion-corrected cortical surface and multiplying cortical magnification factor with RF size. The point image size was larger in central vision than in peripheral vision, indicating that projection from RGCs to V1 is more divergent in the central visual field. The divergent projections from central visual field can occur at several levels of the visual pathway; the projection from retina to LGN, the projection from LGN to the input layer of V1 (i.e., layer IV), or intracortical projections within V1 (e.g., layer IV to layer III). The divergence of projections can be quantified by calculating the ratio of magnification factors at two successive stages (e.g., the V1 magnification factor divided by the LGN magnification factor). These ratios have been obtained experimentally, with that for the LGN vs. retina being 3.5 times higher in central vision than in peripheral vision (Connolly and Van Essen, 1984; Adams and Horton, 2003). Similarly, the ratio of V1 vs. LGN is six times higher for central visual fields than for those in the periphery (Adams and Horton, 2003). These findings indicate that divergent projections underlying the large cortical representation of central visual fields occurs both at the levels of the LGN and V1.

**Figure 1:**
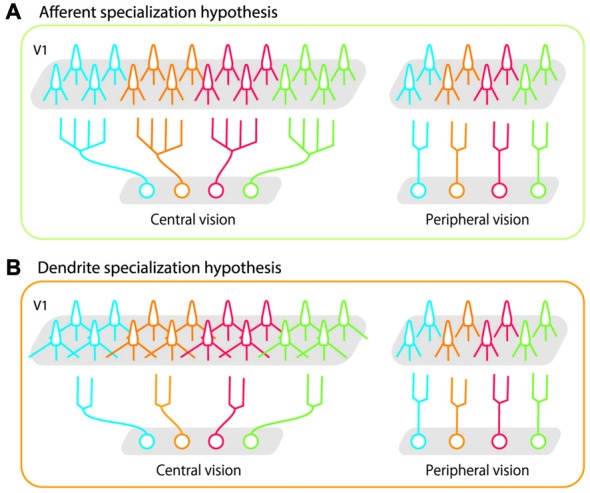
**Schematic illustrations of possible bases for high foveal representation in the cortex. (A)** The afferent specialization hypothesis. Axons of projecting neurons cover a wider area in central vision than in peripheral vision.** (B)** The dendrite specialization hypothesis. Dendritic arbors of target neurons (in this case, in primary visual cortex (V1)) cover a wider area in central vision than in peripheral vision. In both cases, each neuron sends its signals to a larger number of V1 neurons (twice the number of neurons than periphery) if they represent central vision than if they represent peripheral vision. As represented in gray shaded areas in V1, the greater magnification in central vision is implemented in both cases. Our results demonstrated that the morphology of layer-III pyramidal neurons in V1 was uniform across the retinotopic map, and that dendritic specialization did not occur in these cells.

Two potential neural mechanisms can explain these divergent projections. In one scenario, afferent axon terminals of neurons that represent the central visual field branch more extensively and make subsequent synaptic connections with target neurons in a wider area than do those representing peripheral visual fields (Figure 1A, afferent specialization hypothesis). In the other scenario, the amount of axon branching is constant across eccentricities, but neurons representing the central visual field have longer dendritic arbors with more branching than those representing the peripheral visual fields (Figure 1B, dendrite specialization hypothesis). This would allow neurons distributed over a wider cortical surface share the same afferent inputs in the foveal region than in the peripheral region. Both these scenarios can be implemented in the LGN and in V1 (layer IV and layer II-III). Florence and Casagrande (1987) demonstrated that the first scenario is implemented in layer IV of V1; the geniculo-striate axons of neurons carrying information from the central visual field branch more extensively than those from the peripheral visual fields. No studies have yet tested either scenario in layer III of V1. In the present study, we tested the second scenario by examining dendritic morphology of layer III pyramidal neurons at various eccentricities in macaque V1. We used intracellular dye injection to examine whether dendritic morphology of V1 neurons differed across a wide region in the retinotopic map representing 0–20° visual eccentricity. Our results showed that dendritic extent, branching complexity, spine density and total number of spines were similar across layer-III neurons, regardless of their eccentricity. This indicates that the geometrical sampling of these neurons is uniform in this portion of the retinotopic map. We suggest that the large cortical area devoted to central vision is not achieved by the dendritic morphology in layer-III pyramidal neurons of V1, but rather by processes at the preceding sites along the retino-geniculo-striate pathway.

## Materials and Methods

### Animals and Care

Three male cynomolgus macaques (*Macaca fascicularis*) aged 4.5–7.5 years were used in the experiments. All were raised at Shiga Medical School (Otsu, Shiga, Japan; Table 1). The animal experiment committee of Osaka University (Suita, Osaka, Japan) approved the protocols for animal care and experimentation, which were conducted in accordance with the *Guide for the Care and Use of Laboratory Animals* of the National Institutes of Health (DHEW Publication No. (NIH) 85–23, Revised 1996, Office of Science and Health Reports, DRR/NIH, Bethesda, MD 20205, USA).

**Table 1:** **Animals and number of sampled primary visual cortex (V1) neurons**.

					Number of labeled neurons		
Name	Body weight (kg)	Age (year)	Hemisphere	Sex	0°	1°	20°
CI11	6.4	7.5	Left	Male	–	20	19
CI12	2.7	4.5	Left	Male	20	5	25
CI14	3.3	4.5	Left	Male	32	–	–
	–	–	Right	–	70	11	–
Total number of neurons					122	36	44

### Electrophysiological Mapping of Retinotopic Organization

One monkey (CI14) was used for mapping the retinotopic organization of V1 electrophysiologically before intracellular dye injection. The animal underwent aseptic surgery for the placement of a plastic post on the skull for head restraint. The monkey was first premedicated with atropine sulfate (0.1 mg/kg administered intramuscularly [i.m.]); Mitsubishi Tanabe Pharma, Osaka, Japan) to reduce salivation and to promote sedation during surgery. It was then sedated with ketamine HCl (Ketalar®, 25 mg/kg administered i.m.; Sankyo, Tokyo, Japan). Surgical anesthesia was accomplished with isoflurane (Forane®, 0.5–2% in a mixture of 70% nitrous oxide and 30% oxygen; Abbott Japan, Tokyo, Japan). The local anesthetic lidocaine (AstraZeneca, London, UK) was applied to pressure points or incision sites before mounting the monkey in the stereotaxic instrument or making incisions. After exposing the top of the skull with a scalpel blade, the head post was fixed with acrylic resin to four stainless steel bolts inserted into the skull. Throughout the surgery, heart rate, exhaled carbon dioxide (ECO_2_), and peripheral oxygen saturation (SpO_2_) were continuously monitored, and body temperature was maintained near 37°C with a heating pad. After surgery, the monkey was treated with the antibiotic cefotiam hydrochloride (Pansporin®, 8 mg/kg administered i.m.; Takeda Pharmaceutical, Osaka, Japan), the analgesic ketoprofen (Menamin®, 0.8 mg/kg administered i.m.; Sanofi Aventis, Tokyo, Japan), and the corticosteroid dexamethasone sodium phosphate (Decadron®, 0.1 mg/kg administered i.m.; Banyu Pharmaceutical, Tokyo, Japan).

After a recovery period of 1 week, we examined eyes with a keratometer (KR-7100, Topcon, Tokyo, Japan) to select appropriate contact lenses that allowed images at a distance of 114 cm to be focused on the retina. Photographs of the retinal fundus were taken with a retinal camera (TRC-50X, Topcon) to determine the positions of the optic disc and area centralis (see Wang et al., 2002, for further details).

After another week, we performed an electrophysiological experiment to determine the retinotopic map in the right hemisphere of V1. The monkey was initially anesthetized as described above. Then, during the recording session, isoflurane and nitrous oxide were removed, and the monkey was infused with fentanyl citrate (Fentanest®, Daiichi-Sankyo, Tokyo, Japan; 0.035 mg/kg/h), and immobilized with pancuronium bromide (Mioblock®, Organon Japan, Osaka, Japan; 0.02 mg/kg/h; Popilskis and Kohn, 1997). The lactated Ringer solution with 5% glucose (Terumo, Tokyo, Japan) for infusion (10 ml/h) contained atropine sulfate (0.01 mg/kg/h), an antibiotic (Pentcillin®, Toyama Chemical, Toyama, Japan; 0.04 mg/kg/h, intravenous [i.v.]), and riboflavin (Bisulase®, Toa Eiyo, Tokyo, Japan; 0.8 mg/kg/h, i.v.). The pupils were dilated and the lenses were relaxed by applying 0.5% tropicamide, 0.5% phenylephrine hydrochloride (Mydrin®-P, Santen, Osaka, Japan). The corneas were covered with contact lenses of appropriate refractive power and curvature with an artificial pupil (diameter, 3 mm) to focus on a tangent screen placed 114 cm away. The center of the screen was aligned to the projection of the fovea.

**Figure 2:**
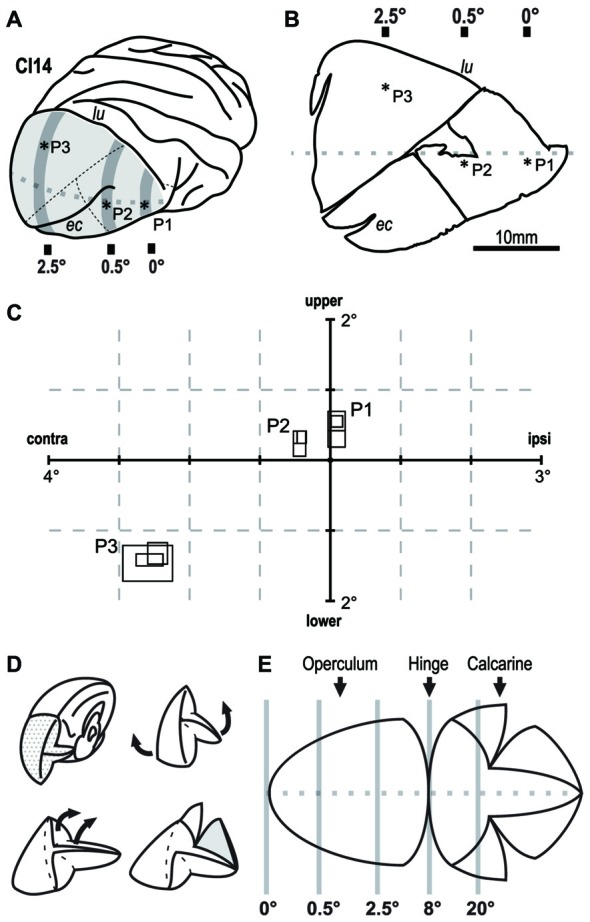
**Retinotopic mapping of the V1 in monkey CI14.** Extracellular single-unit recording revealed the retinotopy of V1 in CI14. **(A)** Asterisks indicate the points penetrated on the brain surface. **(B)** Flat-mounted tissue for dye injection. Asterisks indicate the tracks on the slice tissue. **(C)** The size and location of the receptive field (RF) in the visual field. The three overlapping rectangles at each penetration (P1–P3), indicate RFs recorded from different depths along the penetration. lu: lunate sulcus. ec: ectocalcarine sulcus. **(D,E)** Schematic drawings that show how the V1 tissue was unfolded and flattened. Gray lines indicate approximate eccentricity lines on V1.

We extracellularly recorded single-cell activity using tungsten electrodes (impedance: 0.5–1.2 MΩ at 1 kHz; FHC, Bowdoin, ME, USA). A motorized micromanipulator (PC-5N; Narishige, Tokyo, Japan) was used to control the electrodes. After isolating action potentials from a single neuron, we mapped the position and size of its RF using light-bar stimuli. Bar stimuli of varying lengths and orientation were projected from a retinoscope onto a white screen placed 114 cm from the eyes in a dark environment. For each penetration, we recorded three cells at an interval of 300 μm (Figures 2A–C). After completing the physiological recording, the monkey was deeply anesthetized with sodium pentobarbital, perfused with fixative and buffered saline, and subjected to dye injection experiment (see the next section for details).

### Intracellular Dye Injection

The intracellular dye injection techniques and immunohistochemical procedures have been described in detail elsewhere (Elston and Rosa, 1997; Elston et al., 2010). Briefly, animals were overdosed with sodium pentobarbital (Nembutal®, >75 mg/kg, administered intravenously; Dainippon Sumitomo Pharma, Osaka, Japan) and perfused intracardially with 0.1 M potassium phosphate buffer (PB) saline (PBS, pH 7.2) and 4% paraformaldehyde (Merck, Kenilworth, NJ, USA). Tissue blocks were taken from the occipital lobe including V1. We removed white matter from the blocks, and unfolded the remaining gray matter (Figures 2D,E). The unfolded gray matter was flattened by sandwiching it between two glass-slides, and was postfixed overnight in a solution of 4% paraformaldehyde in 0.1 M PB.

We cut the flattened gray matter into sections tangentially to the cortical surface with the aid of a vibratome (Vibratome® Series 1500; The Vibratome Company, St. Louis, MO, USA). We then cut the blocks alternately into 250-μm and 50-μm thicknesses. The 250-μm thick sections were used for fluorescent dye injection. We used the 50-μm sections for staining Nissl substance with Cresyl violet to visualize neuronal cell-bodies and determine the cortical layers. For the 250-μm thick sections for dye injection we first stained cellular nuclei by soaking them in a solution containing 10^−5^ M of 4,6-diamidino-2-phenylindole (DAPI, D9542; Sigma-Aldrich, St. Louis, MO, USA) in PB at room temperature for 3 min. The section was then placed between Millipore filters (AABG02500; EMD Millipore, Billerica, MA, USA). The Millipore filter above the section had a 6-mm in diameter circular hole to allow microelectrode access to the section. The section was then immersed in PBS and mounted in a custom-made plastic dish on a fixed-stage microscope (Eclipse FN1; Nikon, Tokyo, Japan), and was illuminated with light for ultraviolet–blue excitation (380–420 nm). Under visual guidance, we impaled DAPI-labeled neurons and applied a negative voltage to the microelectrode (up to 20 nA; Dual Microiontophoresis Current Generator, World Precision Instruments, Inc., FL, USA) to generate a continuous current for injecting Lucifer Yellow (Lucifer Yellow CH dilithium salt, L-0259, dissolved in 0.05 M Tris buffer; Sigma-Aldrich).

**Figure 3:**
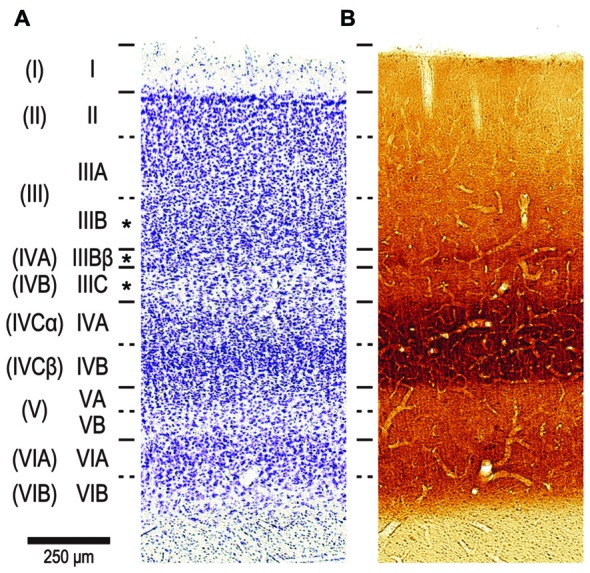
**The nomenclature of layers in V1 by Brodmann (1909) and Hassler (1966).** Two consecutive sections stained by Cresyl violet for Nissl substance **(A)** and cytochrome oxidase (CO) histochemistry **(B)**. The sections were from a 2-year old, male cynomolgus macaque, and were aligned to each other by referring to blood vessels. The layers by Hassler’s nomenclature are listed left, while Brodmann’s divisions are shown far left in parenthesis. The original layer IVC by Brodmann was later divided into sublayers IVCα and IVCβ (Polyak, 1957; Lund, 1973). We injected neurons in Hasslers’s layer III, which contains Brodmann’s layer IVA and IVB in addition to layer III (see asterisks in left).

We focused our analysis on pyramidal neurons in the lower part of layer III. For reasons outlined elsewhere (Casagrande and Kaas, 1994; Elston and Rosa, 1998), we used the nomenclature of Hassler (1966) for the cortical layer. Hassler’s layer III includes layers IVA and IVB in addition to layer III as characterized by Brodmann (1909) (Figures 3A,B; Balaram et al., 2014). We were able to unambiguously distinguish between layer III and layer IV in the DAPI-labeled sections under fluorescent microscope because neurons are denser and smaller in layer IV than in layer III (See Figure 3 of Elston and Rosa, 1997). We aimed electrodes to neurons with a round nucleus (stained with DAPI) immediately above the granular cell layer. All these neurons turned out to be pyramidal neurons; they possessed a stump of an unambiguous apical dendrite, and exhibited dendritic spines. Our sample may thus include a mixed population of pyramidal neurons from layers IVA, IVB and IIIB of Brodmann, but not non-pyramidal neurons such as spiny stellate cells and inhibitory interneurons (see asterisks in Figure 3).

After completing injections in a sufficient number of neurons, we processed the section to generate a light-stable reaction product (Elston and Rosa, 1997). The sections were immersed in a solution (2% bovine serum Albumin [A3425; Sigma-Aldrich], 1% Triton X-100 [Sigma-Aldrich], 0.1% sodium azide, and 5% sucrose in PB) containing 0.6 μg/mL biotinylated anti-Lucifer yellow (A-5751; Invitrogen, Carlsbad, CA, USA) for 4–11 days at room temperature to let the antibody infiltrate into thick tissues (250 μm). They were then washed three times for 10 min each in PB, incubated in streptavidin–biotinylated horseradish peroxidase complex (1:100, RPN1051; GE Healthcare, Little Chalfont, Buckinghamshire, UK) for 2 h, and washed three times for 10 min each in PB. We put the tissue into 1% hydrogen peroxide in 0.1M PB for 5 min. This process helped keep the background staining minimum. After washing the sections three times for 10 min each in PB, they were incubated in 0.5% 3, 3′-diaminobenzidine tetra-hydrochloride (DAB, D5637, 1:200 in PB; Sigma-Aldrich) for 10 min at room temperature. The sections were finally reacted in a solution containing 1% hydrogen peroxide and 0.5% DAB in PB. This method yields a robust, light-stable reaction product.

### Morphological Analysis

We selected neurons for analysis only when their basal dendritic arbors were fully contained within the tissue section. They were reconstructed with Neurolucida software (MBF Bioscience, Williston, VT, USA) coupled to a microscope (Eclipse 80i; Nikon) that was equipped with a motorized stage (Ludl Electric Products, Hawthorne, NY, USA) and a charge-coupled device camera (CCD; CX9000; MBF Biosciences).

The dendritic field area was determined in the tangential plane as the area contained within a convex hull traced around the outermost distal dendritic terminations in reconstructions collapsed to yield two-dimensional (2D) images (see Figure 7A inset). The cell-body area and total dendritic length were also calculated from these 2D projections for compatibility with previous studies on primate V1 (Elston et al., 1996, 2009, 2010; Elston and Rosa, 1997, 1998). The branching profiles of dendritic trees were determined by Sholl (1953) analyses. In this analysis, we counted intersections between the dendritic arbor and concentric circles. The circles had their center on the cell body with varying radii incremented at 10-μm step. By plotting the number of intersections against the radii, we obtained the entire Sholl profile for a neuron. The profile visualizes branching-point location and dendritic extent.

**Figure 4:**
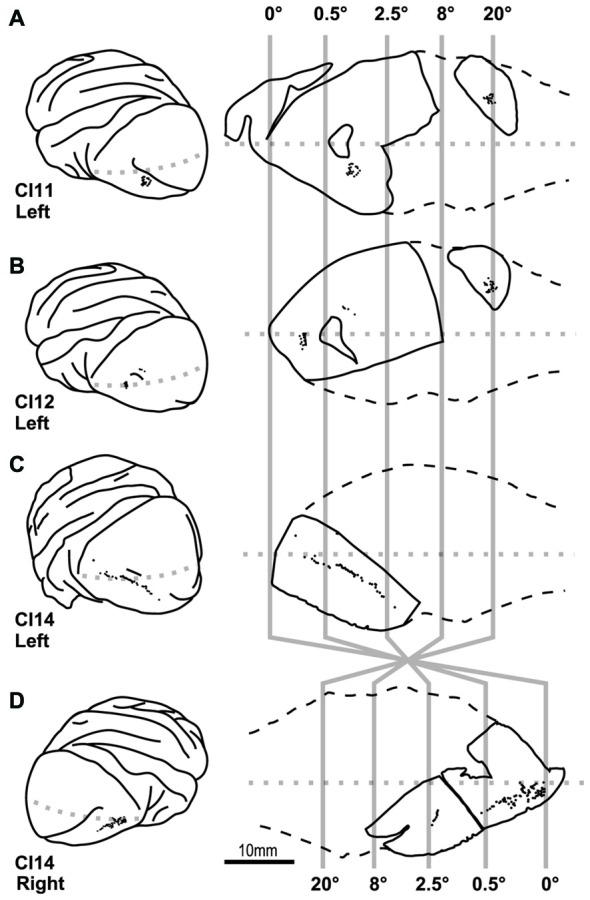
**Location of cells labeled by intracellular dye injection.** Dye-injected cells are plotted on the lateral surface of the cortex (left) and on slice surfaces (right). Vertical gray lines indicate boundaries for estimated eccentricities of 0°, 0.5°, 2.5°, 8° and 20°. **(A–D)** Indicate results from four hemispheres of three monkeys (CI11, 12 and 14).

**Figure 5:**
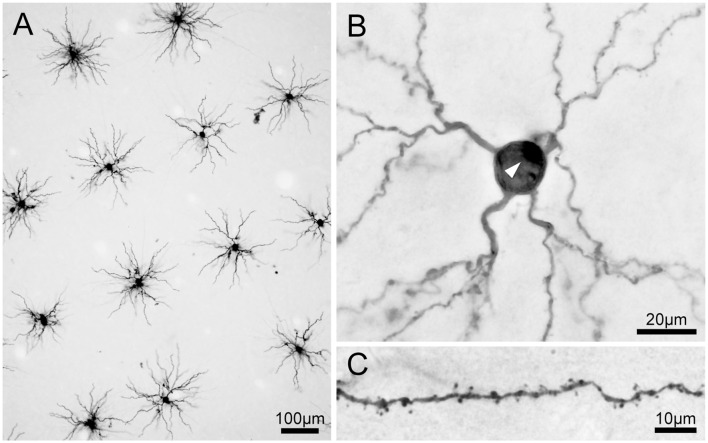
**Photomicrographs of 3,3′-diaminobenzidine tetrahydrochloride (DAB) product of Lucifer Yellow-injected pyramidal neurons.** **(A)** A low-power photomicrograph of dye-injected pyramidal neurons. Neurons were selected for injections with adequate spacing so that labeled dendrites did not overlap between adjacent labeled neurons. Most of the basal dendrites of the pyramidal neurons are included in the 250-μm thick tangential section. **(B)** A pyramidal neuron viewed at high magnification. The white arrowhead indicates the stump of the apical dendrite truncated at the slice surface. **(C)** Dendritic spines are clearly visible on dendrites.

**Figure 6:**
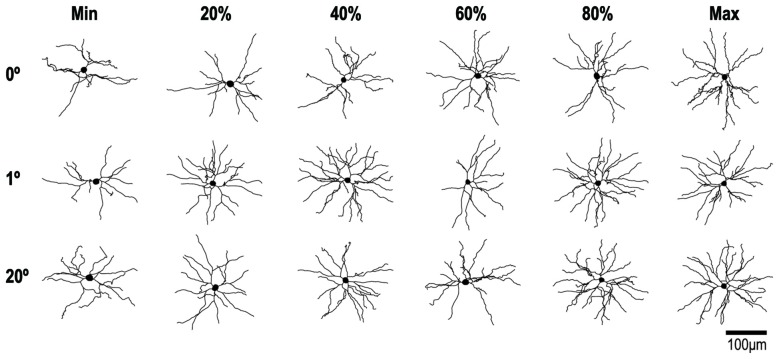
**Reconstructions of representative layer-III pyramidal neurons with RFs at 0°, 1° and 20° of eccentricity.** Cells were viewed in the plane tangential to the cortical surface. The neuron with the smallest basal dendritic field area at each location is illustrated on the left of each row and the neuron with the largest is illustrated on the right. The other neurons in each row represent 20% increments in dendritic field area (i.e., the 20th, 40th, 60th and 80th percentiles).

**Figure 7:**
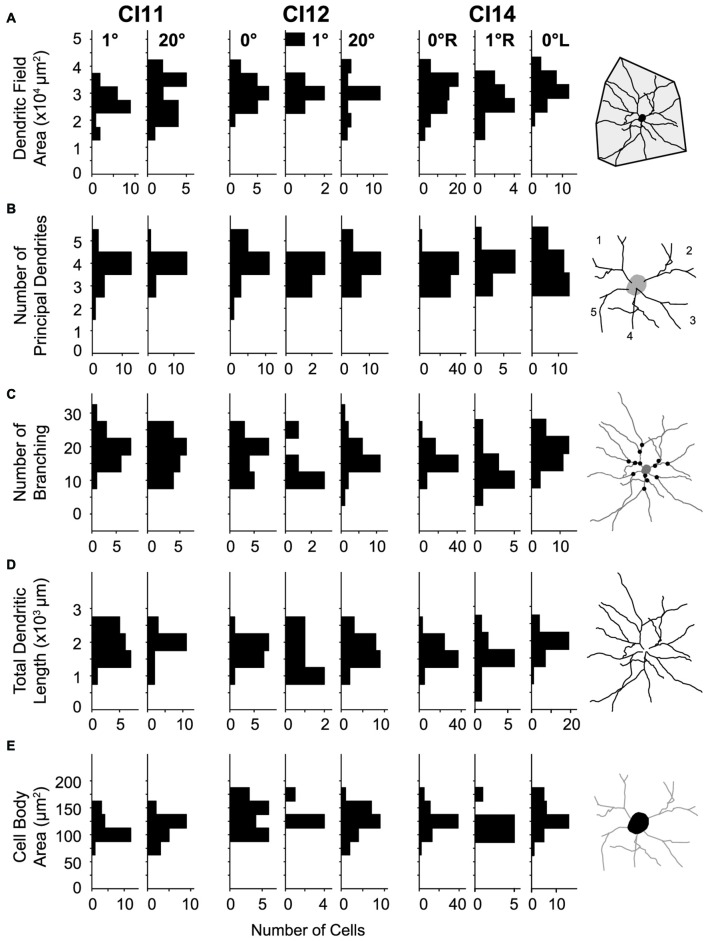
**Distributions of morphological parameters for layer-III pyramidal neurons.** **(A)** Dendritic field area measured as an area of a convex hull over dendrites. **(B)** Number of principal dendrites. **(C)** Number of branches represented by the number of bifurcations of dendritic arbors. **(D)** Total length of dendrites. **(E)** Cell-body area. All parameters were measured from two-dimensional projections of reconstructed cells.

Dendritic spines are loci for excitatory synapses (Gray, 1959; DeFelipe et al., 1988; Arellano et al., 2007). We determined the profile of spine densities along a dendrite by counting the number of spines per 10-μm segment using a 100× oil-immersion objective with correction collars (numeric aperture: 1.49; CFI Apo TIRF 100× H/1.49, Nikon; Valverde, 1967). The objective lens with correction collars can focus deeper in the tissue than a lens without correction collars. This is important for minimizing underestimation of spine counts; stubby spines only slightly protruded from dendritic shafts, and even mushroom spines were sometimes too crowded to dissociate from each other. For spine counting, we selected dendrites extending parallel to the section to avoid trigonometric errors.

We estimated the total number of spines in the basal dendritic tree of an “average” cell at three representative retinotopic locations (0°, 1°, 20°) by calculating the product of the average number of dendrites and the average spine density for corresponding segments along the dendrites (Elston, 2001).

We estimated shrinkage of the brain tissue caused by perfusion in another adult monkey. Before perfusion, we implanted three pins into the brain at 4.00 mm intervals. After perfusion with the PB and paraformaldehyde using the same protocol as in the main experiments, we measured the distances between pins. The distances (between pin 1 and pin 2, and between pin 2 and pin 3) were both 3.89 mm (2.5% shrinkage). Given the small degree of shrinkage, we made no shrinkage correction for the measured values.

## Results

We visualized 202 layer-III pyramidal neurons in varying locations within the V1 from four hemispheres of three monkeys to examine whether their basal dendritic morphology differed depending on their location in the retinotopic map (Figure 4). We reconstructed the basal dendritic trees of these neurons to the full extent of their projections (Table 1). We then analyzed the density and number of dendritic spines by counting 18, 496 individual dendritic spines. As we demonstrate below, the dendritic morphology of layer-III pyramidal neurons was uniform across 0–20° representation in the retinotopic map of V1.

### Location of Dye-Injected Neurons in The Retinotopic Map of V1

In V1 of the macaque monkey, the foveal region is represented in the anterior ventral part near the tip of lunate sulcus, a parafoveal field of ~8° is represented over the occipital operculum, and a visual eccentricity of 20° is represented within the calcarine sulcus (Figure 2E; Daniel and Whitteridge, 1961; Gattass et al., 1981; Van Essen et al., 1984). We verified this organization by mapping RFs in one case (CI14) before dye injection experiments. We recorded extracellular activity from individual V1 neurons in three widely spaced penetrations (P1-P3) over the occipital operculum (Figures 2A,B). P1 was near the anterior end of the operculum close to the tip of the lunate sulcus. P2 was in the middle of the operculum just below the extracalcarine sulcus, and P3 was near the mid-sagittal edge of the operculum. We determined the RFs of three neurons in each of the three penetrations (Figure 2C). The horizontal eccentricities of the RFs in these penetrations were 0° (P1), 0.5° (P2) and 2.5° (P3). The mean RF size for each penetration was 0.115 deg^2^ (P1), 0.077 deg^2^ (P2) and 0.357 deg^2^ (P3; Figure 2C). The visual field representation and the RF-eccentricity relationship were consistent with those reported previously.

Our dye injections were aimed to three regions; one near the tip of the lunate sulcus that represents 0–0.5° (0° group), one in the middle of the occipital operculum that represents 0.5–2.5° (2° group), and one in the calcarine sulcus that represents >20° (20° group; Figure 4). In the following analyses, we compared the dendritic and somal morphology between these regions.

### Morphology of Basal Dendrites and Cell Bodies in The Primary Visual Cortex

Viewed from above, labeled neurons in tangential sections exhibited radially projecting basal dendrites (Figure 5A). We injected neurons with enough spacing so that the labeled dendrites of one neuron did not overlap with those from neighboring neurons. We were also careful to inject neurons whose cell bodies were located a few tens of microns below the surface of the section. We took this precaution to ensure that most of the labeled dendrites were entirely contained within the section. All labeled neurons were unambiguously identified as pyramidal neurons from their characteristic basal dendrites, a stump of a thick apical dendrites projecting towards the surface of the slice (i.e., toward the cortical surface; Figure 5B), and numerous spines along the dendrites (Figure 5C).

Figure 6 shows examples of the reconstructed dendritic morphology of the labeled neurons. For 0°, 2° and 20° groups, neurons are lined up separately with the smallest basal dendritic field area on the left (Min) and the largest on the right (Max). In between are neurons whose dendritic field areas fall in the 20th, 40th, 60th and 80th percentiles. The extent, number and branching complexity appear strikingly similar across the different eccentricity groups.

For quantitative comparison, we measured basal dendritic field area, number of principal dendrites, number of branches, total dendritic length and cell-body area. We analyzed these morphometric parameters for the following reasons. The dendritic field size critically influences the geometrical range of input sampling (Malach, 1994). The total dendritic length determines the availability for synaptic contacts (Gray, 1959). The number of principal dendrites and number of branchings determine the number of components for dendritic computation (Spruston, 2008).

The basal dendritic fields of neurons in V1 were similar across visual eccentricities (*p* = 0.012, Kruskal-Wallis test; mean ± SD: 3.11 × 10^4^ ± 0.65 × 10^4^ μm^2^ [*n* = 122], 2.76 × 10^4^ ± 0.64 × 10^4^ μm^2^ [*n* = 36] and 2.92 × 10^4^ ± 0.77 × 10^4^ μm^2^ [*n* = 44] for the 0°, 1° and 20° groups, respectively; Figure 7A). The number of principal dendrites was also similar across groups (*p* = 0.21; 3.7 ± 0.65, 3.78 ± 0.64 and 3.89 ± 0.58; Figure 7B). Similarly, neither the number of dendritic branchings (*p* = 0.71; 19 ± 4, 19.3 ± 6.2 and 19.8 ± 5.3; Figure 7C) nor the total length of dendrites (*p* = 0.23; 1.81 × 10^3^ ± 0.41 × 10^3^ μm, 1.76 × 10^3^ ± 0.54 × 10^3^ μm and 1.93 × 10^3^ ± 0.5 × 10^3^ μm; Figure 7D) differed across groups.

We applied Sholl analysis to 2D reconstructions of the labeled neurons to analyze their dendritic branching geometry (Sholl, 1953). The Sholl profile of the neuron indicates the spatial distribution of dendritic arbors. The distance at which the number of intersections takes the maximum value indicates the location of the greatest number of dendritic branching.

**Figure 8:**
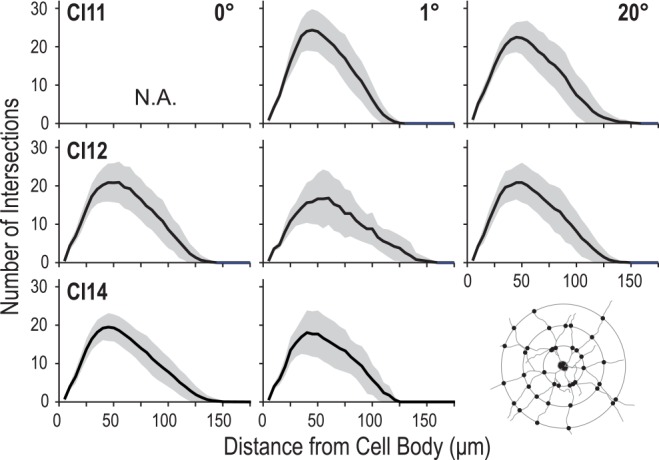
**Profiles of dendritic branching visualized by Sholl analysis.** Each panel shows the average (solid line) and standard deviation (shaded area) of Sholl profiles for a given animal (C11, C12 or C14) at each eccentricity (0°, 1° and 20°).

The number of intersections between dendrites and Sholl rings gradually increased with distance from the cell body, peaked around 50 μm, and then declined slowly towards zero around 120–150 μm. We consistently found similar profiles between the regions and between the animals (Figure 8). The peak number of intersections showed a slight difference between the three regions (*p* = 0.023, Kruskal–Wallis test; 20.7 ± 3.8 for the 0° group, 22.2 ± 6.3 for the 1° group and 22.8 ± 4.7 for the 20° group). The peak value for the 0° group was smaller than that for the 1° group (*p* = 0.01, *post hoc* Mann-Whitney *U*-test). The distance from the cell body at which the peak value occurred did not differ across groups (*p* = 0.62, Kruskal–Wallis test; 43 ± 8.5 μm, 42.5 ± 7.6 μm and 43.8 ± 7.6 μm for the 0°, 1° and 20° groups, respectively).

Cell-body area differed between the three groups (*p* = 0.002; Kruskal-Wallis test; 130 ± 23 μm^2^, 115 ± 22 μm^2^ and 125 ± 29 μm^2^ for the 0°, 1° and 20° groups, respectively; Figure 7E). *Post hoc* analysis showed that cell-body area only differed between the 0° and 1° groups (*p* = 3 × 10^–4^, *post hoc* Mann-Whitney *U*-test), while no significant difference was detected for the other comparisons (*p* > 0.09).

### Dense Sampling of Neurons in the Foveal Representation

So far, our analysis has compared the dendritic morphology between three regions representing widely different visual locations (0°, 1° and 20°). Because changes in visual field magnification are steepest near the foveal region, we might have missed subtle changes, if any, in the dendritic morphology of neurons in the foveal region. In the next experiments, we sampled neurons densely across the visual eccentricity near the fovea to determine if systematic changes occur in the dendritic morphology in this region.

**Figure 9:**
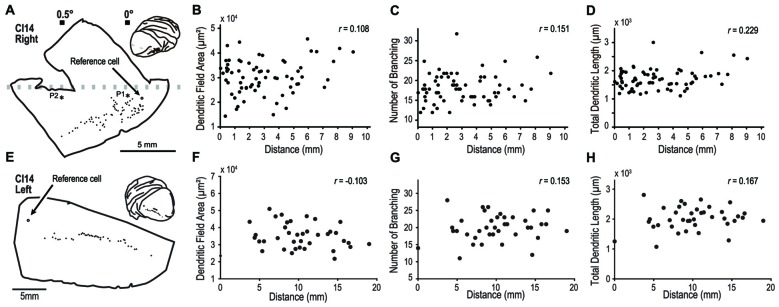
**Analysis of dendritic structure from the fovea to the parafovea.** **(A–H)** indicate results from the right and left hemispheres of CI14, respectively. We injected dye into a large number of pyramidal neurons distributed over a region covering visual fields from the fovea to the periphery to see any specialization in this region. **(A,E)** Each filled circle indicates the location of labeled cells. The open circles are the point-of-reference cells. Asterisks labeled with P1 and P2 indicate the penetration points in the visuotopic mapping experiment (see Figure 2). (**B–D, F–H)** The dendritic field area, number of branching points and total dendritic length are plotted against the distance from the reference cell.

In one experiment, we labeled 70 pyramidal neurons along an 8-mm line from the right hemisphere (Figure 9A). In this animal, we experimentally determined the retinotopic map by prior electrophysiological recording. The labeled neurons were from an estimated eccentricity of 0–0.5°. We plotted dendritic field area, number of branching points and total dendritic length, against the distance from a reference cell located at 0° (open circle, Figure 9A). We found no correlations between this distance and the three morphological features; dendritic field area (Pearson’s linear correlation coefficient *r* = 0.108, *p* = 0.14; Figure 9B), number of branching points (*r* = 0.151, *p* = 0.21; Figure 9C), and total dendritic length (*r* = 0.229, *p* = 0.030, Figure 9D). In the other experiment, we visualized 40 cells in the left hemisphere of CI14 (Figures 9E–H) along a 20-mm line. In this hemisphere, we did not measure the retinotopic map with electrophysiology. Again, there were no correlations between the cortical location and dendritic structure (*r* = −0.103, 0.153 and 0.167, *p* = 0.531, 0.354 and 0.302 for dendritic field area, number of branching points, and total dendritic length, respectively).

Thus, even dense sampling of neurons near the foveal region did not reveal any systematic changes in the basal dendrite morphology of layer-III pyramidal neurons.

### Density and Total Number of Spines on Basal Dendrites

**Figure 10:**
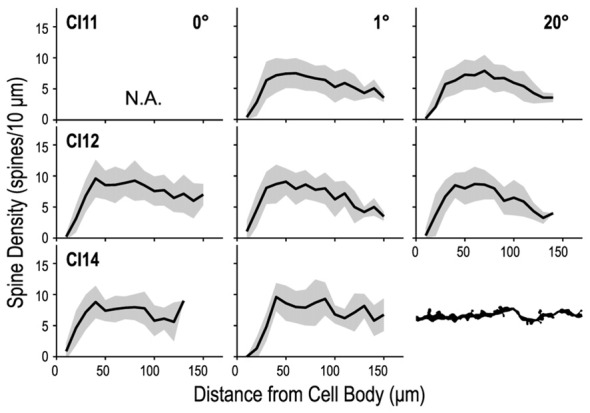
**Density profiles of spines along a dendrite.** The dendrite was divided into 10-μm segments, and spine density was calculated for each segment and tallied along the dendrite. Dendritic spine density peaks at 40–50 μm from the cell body and decreases toward the distal tip.

We counted dendritic spines to determine whether their density differed between the three regions of V1 (representing 0°, 1° and 20°). We counted the numbers of spines in 10-μm segments along the basal dendrites and plotted them against the distance from the cell body (Figure 10). These spine distribution profiles were consistent across the three regions. The initial segment closest to the cell body was devoid of spines. The number of spines steeply increased at the next few segments, and reached a peak around 50 μm from the cell body. Dendritic spine density measured for the entire dendritic length did not significantly differ between the three regions (*p* = 0.34, Kruskal–Wallis test; 6.86 ± 3.42 spines/10-μm, 6.93 ± 3.78 spines/10-μm and 6.74 ± 3.89 spines/10-μm for the 0°, 1° and 20° groups, respectively).

**Figure 11:**
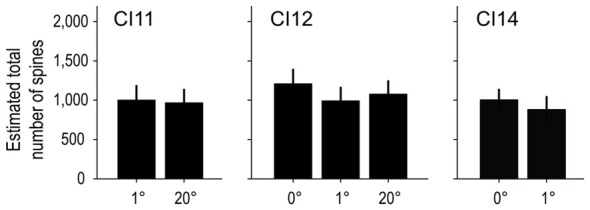
**Estimated total number of spines on an “average” cell.** The total number of spines in the basal dendritic trees of “average” pyramidal neurons was estimated from the product of dendritic branching (Sholl profile) and spine-density profiles. The Sholl profile and spine-density profile used to calculate the total number of spines are the averages over sampled neurons of corresponding subgroups and individual animals. The estimated values were similar across groups.

We calculated the total number of spines in an “average” cell by calculating the product of dendritic length and spine density (the dot product of the Sholl profile and the spine density profile; Elston, 2001). On average, basal dendrites of layer-III pyramidal neurons had 1, 119 ± 158 spines at 0°, 1, 109 ± 205 at 1° and 1, 196 ± 199 at 20°. The estimated values for each area/monkey are plotted in Figure 11. These values were similar between groups (*p* = 1.00 for 0 vs. 1° groups, *p* = 1.00 for 1 vs. 20° groups, and *p* = 0.64 for 0 vs. 20° groups; random permutation test; Bonferroni-corrected).

### Dendritic Morphology of Layer-III Pyramidal Neurons in Area V4

**Figure 12:**
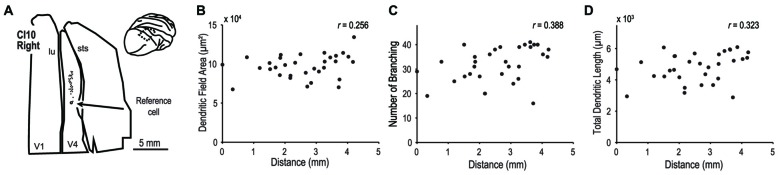
**Morphology of basal dendrites of area V4 neurons.** We injected dye into layer-III pyramidal neurons of V4 in the prelunate gyrus. **(A)** Each filled circle indicates the location of labeled cells. The open circle is the point-of-reference cell. **(B–D)** The dendritic field area, number of branching points and total dendritic length are plotted against the distance from the reference cell. Data are from the case CI10 in Elston et al. (2010).

We extended our analysis to cortical area V4 to investigate whether dendritic morphology in this intermediate stage of the ventral visual pathway (Roe et al., 2012) is similarly consistent across the retinotopic map. We analyzed 32 pyramidal neurons in the dorsal part of V4 located on the prelunate gyrus (Figure 12A inset). The samples used in this analysis were from a previous study (monkey CI10, Elston et al., 2010). We injected dye into neurons along a 6-mm long line on the prelunate gyrus (Figure 12A) so that the labeled neurons encompassed a wide area of visual field representation. The eccentricities of RFs of these neurons were assumed to cover 5° to 15° field representation based on the previously reported retinotopic map in V4 (Gattass et al., 1988; Kolster et al., 2014) and the experiences in our physiological studies (Watanabe et al., 2002; Tanabe et al., 2005; Kotake et al., 2009). Neither dendritic field area, number of branching points, or total dendritic length correlated with the location of the neurons (Figure 12B–D, *r* = 0.256, *p* = 0.158, for dendritic field area; *r* = 0.388, *p* = 0.028, for number of branching points; and *r* = 0.323, *p* = 0.071, for total dendritic length). This suggests that as in V1, the extent and complexity of basal dendrites of layer III neurons do not depend on the RF eccentricity in V4.

## Discussion

We compared basal dendrite morphology of layer-III pyramidal neurons between foveal (0°), parafoveal (1°) and peripheral regions (20°) in the retinotopic map of macaque V1. Morphological characteristics of basal dendrites and their spines, such as dendritic field area, branching and number of dendrites, dendritic length, spine density and total number of spines per neuron were homogeneous across the regions we examined. The area of input sampling and the amount of inputs by a single layer-III neuron via basal dendrites were thus uniform across the 0–20° portion of the retinotopic map. We suggest that the expanded representation of the central visual field in the retinotopic map is accomplished before layer IV neurons project to layer III, and not by specialization of dendritic morphology in layer-III pyramidal neurons.

### Morphological Homogeneity of Dendrites Across Eccentricities

The morphological differences across eccentricities within V1 were much smaller than those between V1 and other visual areas. For example, layer-III pyramidal neurons in V2, V4, and cytoarchitectonic area TEO, have average dendritic field areas 1.2, 1.8, 3.6 times larger than those in V1 (Elston et al., 2010). In contrast, the maximum difference ratio within V1 between the groups representing 0°, 1° and 20° was less than 1.13 (between the 0° and 1° groups). The extensive sampling along lines measuring 20 mm over the surface of V1 did not reveal any systematic changes in morphology of basal dendrites (Figure 9). Layer-III pyramidal neurons in V1 maintain a constant field area of basal dendrites across the cortical surface representing visual field eccentricities from 0 to 20°. This is in sharp contrast to neurons in the retina, which systematically and drastically increase their dendritic field with increased eccentricity (e.g., Wässle et al., 1989 for horizontal cells in macaque; Watanabe and Rodieck, 1989 for ganglion cells in macaque and baboon; Dacey, 1993 for ganglion cells in human).

We did not perform cytochrome oxidase (CO) histochemistry on our samples to determine whether each of the dye-injected neurons was from CO-rich blobs or CO-poor interblobs (Wong-Riley, 1979). Elston and Rosa (1998) previously reported that layer-III pyramidal neurons have a larger basal dendritic field area in CO-blobs (27.0 × 10^3^ μm^2^ on average) than in inter-blob regions (20.1 × 10^3^ μm^2^ on average). This raises a concern that if we sampled neurons with a systematic bias toward blobs or inter-blobs, differences in dendritic morphology across the eccentricities would be canceled out. However, the size and density of CO-blobs is constant within this portion of the retinotopic map of V1 (Farias et al., 1997; Adams and Horton, 2003). Furthermore, the blob size is in the same order as the size of V1 neurons; the diameter of blobs is about 269–281 μm (Farias et al., 1997), and the dendritic field diameter of layer-III V1 neurons is about 200 μm (Elston and Rosa, 1998; Elston et al., 2009; present results). It is therefore unlikely, if not entirely ruled out, that biased sampling occurred in our experiments and affected our conclusion.

The cortical depth of the sampled neurons could also have affected our results because layer II/III pyramidal neurons that are further from the cortical surface have longer basal dendrites (Larkman and Mason, 1990 for rodents). To make sure that the depth of injected cells was comparable among the cells, we selected neurons only immediately above layer IV for injection by confirming the granular appearance of layer IV under the microscope (see “Materials and Methods” Section). We also verified that the next section contained layer IV by staining for Nissl substance with Cresyl Violet. This two-step verification procedure mitigated the artifact due to variation in the cortical depth of injected cells.

### Anatomy of Expanded Central Representation in V1

The central visual representation gradually expands along the retino-geniculo-striate pathway (Perry and Cowey, 1985). Beginning in retina, RGCs are densely packed in the central visual field. RGC density peaks at the fovea and rapidly decreases toward the periphery (Perry and Cowey, 1985; Silveira et al., 1989; Adams and Horton, 2003). Representation of central vision is 3.5 times greater in the LGN than in the retina (Connolly and Van Essen, 1984; Adams and Horton, 2003), and another six times greater in V1 than in the LGN (Adams and Horton, 2003). In this way, the magnified central visual field in V1 results from a series of process along the retino-geniculo-striate pathway.

At least two potential anatomical mechanisms explain the divergent projections. One is that neurons at each earlier region in the pathway arborize their axons more extensively if they represent the central region than if they represent the peripheral regions (Figure 1A: afferent specialization hypothesis). Another is that neurons representing central vision receive broader connections from each earlier stage because of greater dendritic branching (Figure 1B: dendrite specialization hypothesis). In the present study, we tested the dendrite specialization hypothesis along the projection from layer IV to layer III in V1. We demonstrated that across visual eccentricities of 0–20°, layer-III pyramidal neurons in V1 extend their basal dendrites to a similar extent (Figures 7–9) and receive a similar number of inputs within their basal dendritic branches (Figures 10, 11). Thus, dendrite specialization according to eccentricity does not occur in layer III of V1. It remains to be determined whether stellate cells in layer IV exhibit any systematic changes in dendritic morphology across the retinotopic map.

Regarding the axon specialization hypothesis (Figure 1A), there are two possible sites for specialization: LGN axons and layer IV stellate axons. Florence and Casagrande (1987) labeled geniculostriate axons by injecting horseradish peroxidase into nocturnal primate galagos, and found that the axonal arbors spread two times wider in the central visual region than in the peripheral visual region. Whether diurnal primates such as macaques also exhibit afferent specialization needs to be determined. Further, no study has yet compared the spatial extent of axon arborization of layer IV stellate cells between central and peripheral visual representations.

### Cortical Hierarchy vs. Rostrocaudal Position

Pyramidal neurons in layer III of higher cortical areas have a larger and more complex structure than those in lower areas. The higher in the processing hierarchy, the longer the dendrites and the more numerous the branches (Elston and Rosa, 1997, 1998; Amatrudo et al., 2012), and the larger and more extensive distribution of horizontal axon patches (Lund et al., 1993; Fujita and Fujita, 1996; Yoshioka et al., 1996; Tanigawa et al., 2005). Both dendrites and axons of pyramidal neurons change their morphological features postnatally with area-specific growth profiles. The profiles depend on the position of the area in the cortical hierarchy (for macaque monkeys: Elston et al., 2009, 2010; Wang et al., 2016; for marmoset monkeys: Oga et al., 2013; Sasaki et al., 2015). In these analyses, comparison between areas was made without paying attention to retinotopic representations.

Our analysis on V4 (Figure 12) suggests that as in V1, dendritic extent and complexity do not depend on the RF eccentricity in V4. The findings in V1 and V4 together indicate that previously documented differences in basal dendrite morphology between cortical areas did not likely result from unintentional sampling bias from a particular visual eccentricity. Rather, they provide strong support for the claim that the size and branching complexity of layer-III pyramidal neurons differ across different cortical areas (Elston et al., 1996; Elston and Rosa, 1997, 1998; for a review, see Elston and Fujita, 2014).

Although the inter-area difference has often been interpreted to reflect the cortical hierarchy, Elston et al. (1996) raised another possibility that gradual changes in the dendritic arbor may reflect the rostrocaudal position of the labeled neurons. They injected neurons along a rostro-caudal line in the posterior portion of visual cortices in the marmoset. The injected region included secondary visual cortex (V2), the dorsolateral area (DL), and the fundus of the superior temporal area (FST). They found that the gradual change in dendritic size was well fit by a single regression line. We, however, have shown here that neurons sampled from a region covering 20 mm rostrocaudally within V1 were uniform in size and branching of basal dendritic arbors, and in density and total number of spines (Figure 9), suggesting that the cortical hierarchy, rather than the rostro-caudal location, explains the previously reported inter-area differences in dendritic morphology.

### Other Morphological Structures in V1

CO-blobs and ocular dominance columns (ODCs) are prominent anatomical structures observed across the cortical surface of V1. Early studies reported that the size of CO-blobs decreased and the density increased with increasing eccentricity in macaque monkeys (Horton, 1984; Livingstone and Hubel, 1984). However, later studies did not reproduce these findings and showed that the size and density of CO-blob were constant across V1 (macaque monkey, Farias et al., 1997; squirrel monkey, Adams and Horton, 2003). Unlike CO-blobs, ODCs vary their width with eccentricity. ODCs are wider in regions representing central visual field than in regions representing peripheral visual field (LeVay et al., 1985; Horton and Hocking, 1996a, b). On top of this retinotopy-dependent variation, the width of ODCs exhibit striking inter-individual differences (Horton and Hocking, 1996b). When compared between the corresponding retinotopic portions of V1 of different animals, ODCs could exhibit up to a two-fold difference in their width. Basal dendrites of layer III neurons did not exhibit such differences between different retinotopic locations or between individuals. These findings together suggest that layer III pyramidal neurons in the peripheral field may combine binocular inputs more readily than neurons in the central visual field. It would be interesting to compare the distribution of ocular dominance index between the two regions (Hubel and Wiesel, 1962).

Dendrites of spiny stellate neurons in layer IVCα and IVCβ remain in their home ODC where their cell body reside (Katz et al., 1989). Dendrites of pyramidal neurons in layer II/III cross over the CO-blob border (Hübener and Bolz, 1992; Malach, 1992). It is unclear whether dendrites of layer III pyramidal neurons care or ignore, i.e., remain inside or extend over, the border of ODCs. If they do care, peripheral neurons embedded in narrower ODCs would have smaller dendritic diameter than central neurons embedded in wider ODCs. As we showed in the present study, there was no detectable difference across the regions representing 0–20°. Layer III pyramidal neurons likely spread their branches across the border of ODCs. Functional specificity may be substantiated by finer organization of dendritic spines and axonal arborization.

## Conclusion

We present evidence for morphological uniformity of dendrites of layer-III pyramidal neurons across visual eccentricities in V1. Our morphological analysis at the spine level also revealed geometric uniformity in the sampling of synaptic inputs by the basal dendrites of these neurons. The uniform dendritic convergence of information through layers IV to III in V1 suggests that the greater cortical representation of central vision is not ascribable to specialized morphology of pyramidal neurons in layer III, but is likely the result of a cumulative process that occurs earlier in the retino-geniculo-striate pathway.

## Author Contributions

All authors conceived and designed the experiment together. TOg and TOk performed the experiment and analyzed the data. TOg and IF wrote the article.

## Funding

This work was supported by grants to IF from the Japan Science and Technology Agency (Core Research for Evolutional Science and Technology), Ministry of Education, Culture, Sports, Science and Technology (Japan; JP17022025, JP15H01437, JP16H01673, JP16H03384), and Osaka University. TOg was supported by the Japan Society for the Promotion of Science Research Fellowship (JP15J05524).

## Conflict of Interest Statement

The authors declare that the research was conducted in the absence of any commercial or financial relationships that could be construed as a potential conflict of interest.

## References

[B1] Adams D. L. Horton J. C. (2003). A precise retinotopic map of primate striate cortex generated from the representation of angioscotomas. J. Neurosci. 23, 3771–3789. 12736348 10.1523/JNEUROSCI.23-09-03771.2003 PMC6742198

[B2] Amatrudo J. M. Weaver C. M. Crimins J. L. Hof P. R. Rosene D. L. Luebke J. I. (2012). Influence of highly distinctive structural properties on the excitability of pyramidal neurons in monkey visual and prefrontal cortices. J. Neurosci. 32, 13644–13660. 10.1523/JNEUROSCI.2581-12.2012 23035077 PMC3485081

[B3] Arellano J. I. Espinosa A. Fairén A. Yuste R. DeFelipe J. (2007). Non-synaptic dendritic spines in neocortex. Neuroscience 145, 464–469. 10.1016/j.neuroscience.2006.12.015 17240073

[B4] Balaram P. Young N. A. Kaas J. H. (2014). Histological features of layers and sublayers in cortical visual areas V1 and V2 of chimpanzees, macaque monkeys and humans. Eye Brain 2014, 5–18. 10.2147/eb.s51814 25788835 PMC4360995

[B5] Brodmann K. (1909). Vergleichende Lokalisationslehre der Grosshirnrinde. J. Psychol. Neurol. 4, 177–226.

[B6] Capuano U. McIlwain J. T. (1981). Reciprocity of receptive field images and point images in the superior colliculus of the cat. J. Comp. Neurol. 196, 13–23. 10.1002/cne.901960103 7204663

[B7] Casagrande V. A. Kaas J. H. (1994). “The afferent, intrinsic and efferent connections of primary visual cortex in primates,” in Cerebral Cortex, Vol 10. Primary Visual Cortex in Primates, eds Peters A. Rockland K. S. (New York, NY: Plenum), 201–259.

[B8] Chaplin T. A. Yu H.-H. Rosa M. G. P. (2013). Representation of the visual field in the primary visual area of the marmoset monkey: magnification factors, point-image size and proportionality to retinal ganglion cell density. J. Comp. Neurol. 521, 1001–1019. 10.1002/cne.23215 22911425

[B9] Connolly M. Van Essen D. (1984). The representation of the visual field in parvicellular and magnocellular layers of the lateral geniculate nucleus in the macaque monkey. J. Comp. Neurol. 226, 544–564. 10.1002/cne.902260408 6747034

[B10] Cowey A. Rolls E. T. (1974). Human cortical magnification factor and its relation to visual acuity. Exp. Brain Res. 21, 447–454. 10.1007/bf00237163 4442497

[B11] Dacey D. M. (1993). The mosaic of midget ganglion cells in the human retina. J. Neurosci. 13, 5334–5355. 8254378 10.1523/JNEUROSCI.13-12-05334.1993 PMC6576399

[B12] Daniel P. M. Whitteridge D. (1961). “The representation of the visual field on the calcarine cortex,” in The Visual System: Neurophysiology and Psychophysics, eds Jung R. Kornhuber H. (Berlin: Springer-Verlag), 222–228.

[B13] DeFelipe J. Conti F. Van Eyck S. L. Manzoni T. (1988). Demonstration of glutamate-positive axon terminals forming asymmetric synapses in cat neocortex. Brain Res. 455, 162–165. 10.1016/0006-8993(88)90127-8 3416182

[B15] Elston G. N. (2001). Interlaminar differences in the pyramidal cell phenotype in cortical areas 7 m and STP (the superior temporal polysensory area) of the macaque monkey. Exp. Brain Res. 138, 141–152. 10.1007/s002210100705 11417455

[B14] Elston G. N. Fujita I. (2014). Pyramidal cell development: postnatal spinogenesis, dendritic growth, axon growth and electrophysiology. Front. Neuroant. 8:78. 10.3389/fnana.2014.00078 25161611 PMC4130200

[B18] Elston G. N. Oga T. Fujita I. (2009). Spinogenesis and pruning scales across functional hierarchies. J Neurosci. 29, 3271–3275. 10.1523/JNEUROSCI.5216-08.2009 19279264 PMC6666449

[B19] Elston G. N. Oga T. Okamoto T. Fujita I. (2010). Spinogenesis and pruning from early visual onset to adulthood: an intracellular injection study of layer III pyramidal cells in the ventral visual cortical pathway of the macaque monkey. Cereb. Cortex 20, 1398–1408. 10.1093/cercor/bhp203 19846470

[B16] Elston G. N. Rosa M. G. (1997). The occipitoparietal pathway of the macaque monkey: comparison of pyramidal cell morphology in layer III of functionally related cortical visual areas. Cereb. Cortex 7, 432–452. 10.1093/cercor/7.5.432 9261573

[B17] Elston G. N. Rosa M. G. (1998). Morphological variation of layer III pyramidal neurones in the occipitotemporal pathway of the macaque monkey visual cortex. Cereb. Cortex 8, 278–294. 10.1093/cercor/8.3.278 9617923

[B20] Elston G. N. Rosa M. G. Calford M. B. (1996). Comparison of dendritic fields of layer III pyramidal neurons in striate and extrastriate visual areas of the marmoset: a Lucifer yellow intracellular injection. Cereb. Cortex 6, 807–813. 10.1093/cercor/6.6.807 8922337

[B21] Farias M. F. Gattass R. Piñón M. C. Ungerleider L. G. (1997). Tangential distribution of cytochrome oxidase-rich blobs in the primary visual cortex of macaque monkeys. J. Comp. Neurol. 386, 217–228. 10.1002/(sici)1096-9861(19970922)386:2<217::aid-cne4>3.0.co;2-4 9295148

[B22] Florence S. L. Casagrande V. A. (1987). Organization of individual afferent axons in layer IV of striate cortex in a primate. J. Neurosci. 7, 3850–3868. 3121801 10.1523/JNEUROSCI.07-12-03850.1987 PMC6569094

[B23] Fritsches K. A. Rosa M. G. (1996). Visuotopic organisation of striate cortex in the marmoset monkey (*Callithrix jacchus*). J. Comp. Neurol. 372, 264–282. 10.1002/(sici)1096-9861(19960819)372:2<264::aid-cne8>3.3.co;2-o 8863130

[B24] Fujita I. Fujita T. (1996). Intrinsic connections in the macaque inferior temporal cortex. J. Comp. Neurol. 368, 467–486. 10.1002/(sici)1096-9861(19960513)368:4<467::aid-cne1>3.0.co;2-2 8744437

[B25] Gattass R. Gross C. G. Sandell J. H. (1981). Visual topography of V2 in the macaque. J. Comp. Neurol. 201, 519–539. 10.1002/cne.902010405 7287933

[B26] Gattass R. Sousa A. P. Gross C. G. (1988). Visuotopic organization and extent of V3 and V4 of the macaque. J. Neurosci. 8, 1831–1845. 3385477 10.1523/JNEUROSCI.08-06-01831.1988 PMC6569322

[B27] Gattass R. Sousa A. P. B. Rosa M. G. P. (1987). Visual topography of V1 in the Cebus monkey. J. Comp. Neurol. 259, 529–548. 10.1002/cne.902590404 3597827

[B28] Glickstein M. Fahle M. (2000). T. Inouye: visual disturbances following gunshot wounds of the cortical visual area (Translation). Brain 123, 1–101.

[B29] Gray E. G. (1959). Electron microscopy of synaptic contacts on dendrite spines of the cerebral cortex. Nature 183, 1592–1593. 10.1038/1831592a0 13666826

[B30] Hassler R. (1966). “Comparative anatomy of the central visual systems in day- and night-active primates,” in Evolution of the Forebrain, eds Hassler R. Stephan H. (Stuttgart: Thieme Verlag), 419–434.

[B31] Holmes G. Lister W. T. (1916). Disturbances of vision from cerebral lesions, with special reference to the cortical representation of the macula. Brain 39, 34–73. 10.1093/brain/39.1-2.34 PMC2017232 19979442

[B32] Horton J. C. (1984). Cytochrome oxidase patches: a new cytoarchitectonic feature of monkey visual cortex. Philos. Trans. R. Soc. B Biol. Sci. 304, 199–253. 10.1098/rstb.1984.0021 6142484

[B33] Horton J. C. Hocking D. R. (1996a). Anatomical demonstration of ocular dominance columns in striate cortex of the squirrel monkey. J. Neurosci. 16, 5510–5522. 8757263 10.1523/JNEUROSCI.16-17-05510.1996 PMC6578890

[B34] Horton J. C. Hocking D. R. (1996b). Intrinsic variability of ocular dominance column periodicity in normal macaque monkeys. J. Neurosci. 16, 7228–7339. 8929431 10.1523/JNEUROSCI.16-22-07228.1996 PMC6578935

[B35] Hubel D. H. Wiesel T. N. (1962). Receptive fields, binocular interaction and functional architecture in the cat’s visual cortex. J. Physiol. 160, 106–154. 10.1113/jphysiol.1962.sp006837 14449617 PMC1359523

[B36] Hübener M. Bolz J. (1992). Relationships between dendritic morphology and cytochrome oxidase compartments in monkey striate cortex. J. Comp. Neurol. 324, 67–80. 10.1002/cne.903240106 1328331

[B37] Inouye T. (1909). Die Sehstörungen Bei Schussverletzungen Der Kortikalen Sehsphäre Nach Beobachtungen an Verwundeten Der Letzten Japanischen Kriege. Leipzig: Engelmann.

[B38] Katz L. C. Gilbert C. D. Wiesel T. N. (1989). Local circuits and ocular dominance columns in monkey striate cortex. J. Neurosci. 9, 1389–1399. 2703882 10.1523/JNEUROSCI.09-04-01389.1989 PMC6569851

[B39] Kolster H. Janssens T. Orban G. A. Vanduffel W. (2014). The retinotopic organization of macaque occipitotemporal cortex anterior to V4 and caudoventral to the middle temporal (MT) cluster. J. Neurosci. 34, 10168–10191. 10.1523/JNEUROSCI.3288-13.2014 25080580 PMC4115132

[B40] Kotake Y. Morimoto H. Okazaki Y. Fujita I. Tamura H. (2009). Organization of color-selective neurons in macaque visual area V4. J. Neurophysiol. 102, 15–27. 10.1152/jn.90624.2008 19369361

[B41] Larkman A. Mason A. (1990). Correlations between morphology and electrophysiology of pyramidal neurons in slices of rat visual cortex. I. Establishment of cell classes. J. Neurosci. 10, 1407–1414. 2332787 10.1523/JNEUROSCI.10-05-01407.1990 PMC6570081

[B42] LeVay S. Connolly M. Houde J. Van Essen D. C. (1985). The complete pattern of ocular dominance stripes in the striate cortex and visual field of the macaque monkey. J. Neurosci. 5, 486–501. 3973679 10.1523/JNEUROSCI.05-02-00486.1985 PMC6565187

[B43] Livingstone M. S. Hubel D. H. (1984). Anatomy and physiology of a color system in the primate visual cortex. J. Neurosci. 4, 309–356. 6198495 10.1523/JNEUROSCI.04-01-00309.1984 PMC6564760

[B44] Lund J. S. (1973). Organization of neurons in the visual cortex, area 17, of the monkey (*Macaca mulatta*). J. Comp. Neurol. 147, 455–495. 10.1002/cne.901470404 4122705

[B45] Lund J. S. Yoshioka T. Levitt J. B. (1993). Comparison of intrinsic connectivity in different areas of macaque monkey cerebral cortex. Cereb. Cortex 3, 148–162. 10.1093/cercor/3.2.148 8490320

[B46] Malach R. (1992). Dendritic sampling across processing streams in monkey striate cortex. J. Comp. Neurol. 315, 303–312. 10.1002/cne.903150306 1311004

[B47] Malach R. (1994). Cortical columns as devices for maximizing neuronal diversity. Trends Neurosci. 17, 101–104. 10.1016/0166-2236(94)90113-9 7515522

[B48] Myerson J. Manis P. B. Miezin F. M. Allman J. M. (1977). Magnification in striate cortex and retinal ganglion cell layer of owl monkey: a quantitative comparison. Science 198, 855–857. 10.1126/science.411172 411172

[B49] Oga T. Aoi H. Sasaki T. Fujita I. Ichinohe N. (2013). Postnatal development of layer III pyramidal cells in the primary visual, inferior temporal and prefrontal cortices of the marmoset. Front. Neural Circuits 7:31. 10.3389/fncir.2013.00031 23483808 PMC3592264

[B50] Perry V. H. Cowey A. (1985). The ganglion cell and cone distributions in the monkey’s retina: implications for central magnification factors. Vision Res. 25, 1795–1810. 10.1016/0042-6989(85)90004-5 3832605

[B51] Polyak S. (1957). The Vertebrate Visual System. Chicago: University of Chicago Press.

[B52] Popilskis S. J. Kohn D. F. (1997). “Anesthesia and analgesia in nonhuman primates,” in Anesthesia and Analgesia in Laboratory Animals, eds Kohn D. F. Wixson S. K. White W. J. Benson G. J. (New York, NY: Academic), 233–255.

[B53] Roe A. W. Chelazzi L. Connor C. E. Conway B. R. Fujita I. Gallant J. L.. (2012). Toward a unified theory of visual area V4. Neuron 74, 12–29. 10.1016/j.neuron.2012.03.011 22500626 PMC4912377

[B54] Sasaki T. Aoi H. Oga T. Fujita I. Ichinohe N. (2015). Postnatal development of dendritic structure of layer III pyramidal neurons in the medial prefrontal cortex of marmoset. Brain Struct. Funct. 220, 3245–3258. 10.1007/s00429-014-0853-2 25064470

[B55] Schein S. J. de Monasterio F. M. (1987). Mapping of retinal and geniculate neurons onto striate cortex of macaque. J. Neurosci. 7, 996–1009. 3033167 10.1523/JNEUROSCI.07-04-00996.1987 PMC6568992

[B56] Sholl D. A. (1953). Dendritic organization in the neurons of the visual and motor cortices of the cat. J. Anat. 87, 387–406. 13117757 PMC1244622

[B57] Silveira L. C. L. Picanço-Diniz C. W. Sampaio L. F. S. Oswaldo-Cruz E. (1989). Retinal ganglion cell distribution in the cebus monkey: a comparison with the cortical magnification factors. Vision Res. 29, 1471–1483. 10.1016/0042-6989(89)90131-4 2635473

[B58] Spruston N. (2008). Pyramidal neurons: dendritic structure and synaptic integration. Nat. Rev. Neurosci. 9, 206–221. 10.1038/nrn2286 18270515

[B59] Talbot S. A. Marshall W. H. (1941). Physiological studies on neural mechanisms of visual localization and discrimination. Am. J. Ophthalmol. 24, 1255–1264. 10.1016/s0002-9394(41)91363-6

[B60] Tanabe S. Doi T. Umeda K. Fujita I. (2005). Disparity-tuning characteristics of neuronal responses to dynamic random-dot stereograms in macaque visual area V4. J. Neurophysiol. 94, 2683–2699. 10.1152/jn.00319.2005 16000525

[B61] Tanigawa H. Wang Q. Fujita I. (2005). Organization of horizontal axons in the inferior temporal cortex and primary visual cortex of the macaque monkey. Cereb. Cortex 15, 1887–1899. 10.1093/cercor/bhi067 15758199

[B62] Tootell R. B. Silverman M. S. Switkes E. De Valois R. L. (1982). Deoxyglucose analysis of retinotopic organization in primate striate cortex. Science 218, 902–904. 10.1126/science.7134981 7134981

[B63] Valverde F. (1967). Apical dendritic spines of the visual cortex and light deprivation in the mouse. Exp. Brain Res. 3, 337–352. 10.1007/bf00237559 6031165

[B64] Van Essen D. C. Newsome W. T. Maunsell J. H. R. (1984). The visual field representation in striate cortex of the macaque monkey: asymmetries, anisotropies and individual variability. Vis. Res. 24, 429–448. 10.1016/0042-6989(84)90041-5 6740964

[B66] Wang Y. Fujita I. Tamura H. Murayama Y. (2002). Contribution of GABAergic inhibition to receptive field structures of monkey inferior temporal neurons. Cereb. Cortex 12, 62–74. 10.1093/cercor/12.1.62 11734533

[B65] Wang Q. Tanigawa H. Fujita I. (2016). Postnatal development of intrinsic horizontal axons in macaque inferior temporal and primary visual cortices. Cereb. Cortex [Epub ahead of print]. 10.1093/cercor/bhw105 27114175

[B67] Wässle H. Boycott B. B. Röhrenbeck J. (1989). Horizontal cells in the monkey retina: cone connections and dendritic network. Eur. J. Neurosci. 1, 421–435. 10.1111/j.1460-9568.1989.tb00350.x 12106129

[B68] Watanabe M. Rodieck R. W. (1989). Parasol and midget ganglion cells of the primate retina. J. Comp. Neurol. 289, 434–454. 10.1002/cne.902890308 2808778

[B69] Watanabe M. Tanaka H. Uka T. Fujita I. (2002). Disparity-selective neurons in area V4 of macaque monkeys. J. Neurophysiol. 87, 1960–1973. 10.1152/jn.00780.2000 11929915

[B70] Wong-Riley M. (1979). Changes in the visual system of monocularly sutured or enucleated cats demonstrable with cytochrome oxidase histochemistry. Brain Res. 171, 11–28. 10.1016/0006-8993(79)90728-5 223730

[B71] Yoshioka T. Blasdel G. G. Levitt J. B. Lund J. S. (1996). Relation between patterns of intrinsic lateral connectivity, ocular dominance and cytochrome oxidase-reactive regions in macaque monkey striate cortex. Cereb. Cortex 6, 297–310. 10.1093/cercor/6.2.297 8670658

